# Effects of Processing Parameters on the Mechanical, Thermo-Mechanical and Creep-Recovery Properties of Unidirectional Carbon Fiber Reinforced Thermoplastic Polypropylene Composites

**DOI:** 10.3390/polym18111342

**Published:** 2026-05-28

**Authors:** Shaoce Dong, Siwei Xie, Ping Zhou, Puxuan Zhang, Yutan Zhang, Bin Hong, Guijun Xian, Chenggao Li

**Affiliations:** 1 School of Civil Engineering, Harbin Institute of Technology (HIT), 73 Huanghe Road, Nangang District, Harbin 150090, Chinalichenggao@hit.edu.cn (C.L.); 2 School of Civil Engineering, Lanzhou Jiaotong University, No. 88 West Anning Road, Anning District, Lanzhou 730070, China; 3 College of Future Technologies, Hohai University, Changzhou 213200, China; 4 Yangtze River Delta Carbon Fiber and Composite Innovation Center, Changzhou 213126, China

**Keywords:** carbon fiber, polypropylene, manufacturing, mechanical properties, thermo-mechanical, creep-recovery behavior, crystalline behavior

## Abstract

Processing parameters play a key role in the mechanical, thermo-mechanical and creep-recovery properties of unidirectional carbon fiber reinforced thermoplastic polypropylene (CF/PP) composites because of high matrix viscosity, which governs their impregnation and interfacial bonding. This study systematically investigates the effects of molding temperature (190~210 °C), pressure (1~3 MPa), and holding time (5~15 min) on its short beam strength (SBS), storage modulus, loss modulus, tan δ, creep strain, strain recovery, and crystallinity using a Taguchi experimental design. The results presented that processing parameters have a huge effect on CF/PP composites’ SBS, and through the experimental design, the SBS could be improved by 68.1% (21.3~35.8 MPa). Holding time is the most influential parameter for SBS and damping performance, while temperature and pressure interact strongly, highlighting the importance of parameter synergy. There was a strong negative correlation between the crystallinity degree and the SBS of CF/PP composites, and a higher crystallinity degree means a sharper and higher melting peak. Creep-recovery tests reveal near-complete recovery (87~102%) at 30 °C, which decreases to 71~79% at 80 °C due to increased matrix mobility. Finally, it was confirmed that the relatively low SBS of CF/PP composites comes from the void and incomplete matrix impregnation of fibers. The above results advance the design of high-performance, sustainable thermoplastic composites for civil and structural engineering applications.

## 1. Introduction

Fiber reinforced polymer (FRP, fiber and polymer serve as reinforcing phase and matrix, respectively) composites have gained a large-scale application in civil engineering because of their advantages like high strength but low weight, corrosion-resistance and fatigue-resistance [1]. Continuous and unidirectional FRP composites like rebars [2,3] and plates [4] are preferred in civil engineering because their high strength can be fully utilized. According to the report regarding the global market for FRP composites by JEC Observer [5], the construction contributed 33% in the global composite market, but only about 21.2% composites in the construction field are short fiber reinforced polymers.

The matrix of FRP composites mainly includes thermoset and thermoplastic polymers. Thermoset polymers based FRP composites are already widely used in civil engineering, such as epoxy, vinyl-ester and polyurethane [6]. On the other hand, thermoplastic polymers based FRP (FRTP, thermoplastic polymer serves as the matrix of FRP) composites are less used in civil engineering, and according to the data of JEC Observer, FRTP composites are mainly used in automotive and aerospace areas [5]. However, the linear molecular structure of thermoplastic polymers could grant FRTP composites with advantages like secondary-forming [7,8], easier recycling [9,10], welding [11], good durability [12] and better resistance to impacts [13]. Moreover, through the thermal melt recycling method, FRTP composites could serve as a sustainable construction material, which meets the requirements of the development of a sustainable society [14,15]. With the development of manufacturing technologies for FRTP composites, like hot-press [16], thermoplastic pultrusion [17] and tape winding [18], FRTP composites show a promising future in civil engineering fields.

Although FRTP composites have a series of advantages, the high melt viscosity of most thermoplastic polymers becomes a frontier in the perfect impregnation of fibers and, therefore, in producing high-quality FRTP composites. To achieve a better wetting of fibers, generally high temperatures and pressures are required when producing FRTP composites. For instance, Bodaghi et al. [19] illustrated that thermoplastic polymers required much higher processing temperatures than common thermoset polymers. In addition, thermoplastic pultrusion was developed, where the prepreg production is often adopted as the first step, and then prepregs are treated as the feed materials for the final production formation [20]. Moreover, the suitable selection of processing parameters should be determined.

Carbon fiber reinforced thermoplastic polypropylene (CF/PP, carbon fiber and polypropylene serve as the reinforcing fiber and matrix, and abbreviated as CF and PP, respectively) composites could utilize the excellent mechanical properties of carbon fiber [21] and the advantages of polypropylene (PP) like extremely low water absorption and excellent resistance to chemicals [22]. However, nowadays, CF/PP composites investigated in the literature were mostly reinforced by short fibers [23]. Limited studies investigated the manufacturing and mechanical properties of continuous carbon fiber-based CF/PP composites. Sun et al. [24] investigated the influence of stamping temperature (190, 220, 250 °C) on the crystallization behavior, fiber/matrix interface and mechanical properties of woven CF/PP composites. The results showed that higher temperature could improve the crystallinity degree, interface bonding and thermal stability of CF/PP composites. However, over-high temperature could weaken the property of the PP matrix and reduce the mechanical properties of CF/PP composites. Allen et al. [25] found that the thermal processing conditions could change the structure of the crystallization layer along the interface, which affects the tensile properties. The mechanical properties of CF/PP composites not only depend on the fiber content, but also are controlled by the crystal morphology of the interface. Han et al. [26] adopted a coupling agent together with plasma treatment to enhance the interfacial bond between the carbon fiber and the PP matrix. Tian et al. [27] studied the mechanical and electromagnetic interference shielding properties of the PP/carbon fiber/carbon black composite foam bonded with continuous CF/PP prepregs samples. Gabr et al. [28] found that the addition of nano-clay could dramatically enhance the interlaminar fracture toughness of woven CF/PP composites and increase their glass transition temperature. The creep study regarding the creep behavior of continuous CF/PP composites is also quite limited. As an example, Nakada et al. [29] validated the statistical prediction model of the creep failure probability for unidirectional CF/PP composite tapes at different creep stress levels and temperatures. However, the influence of different processing parameters on the creep properties of CF/PP composites has not been investigated yet.

**Figure 1:**
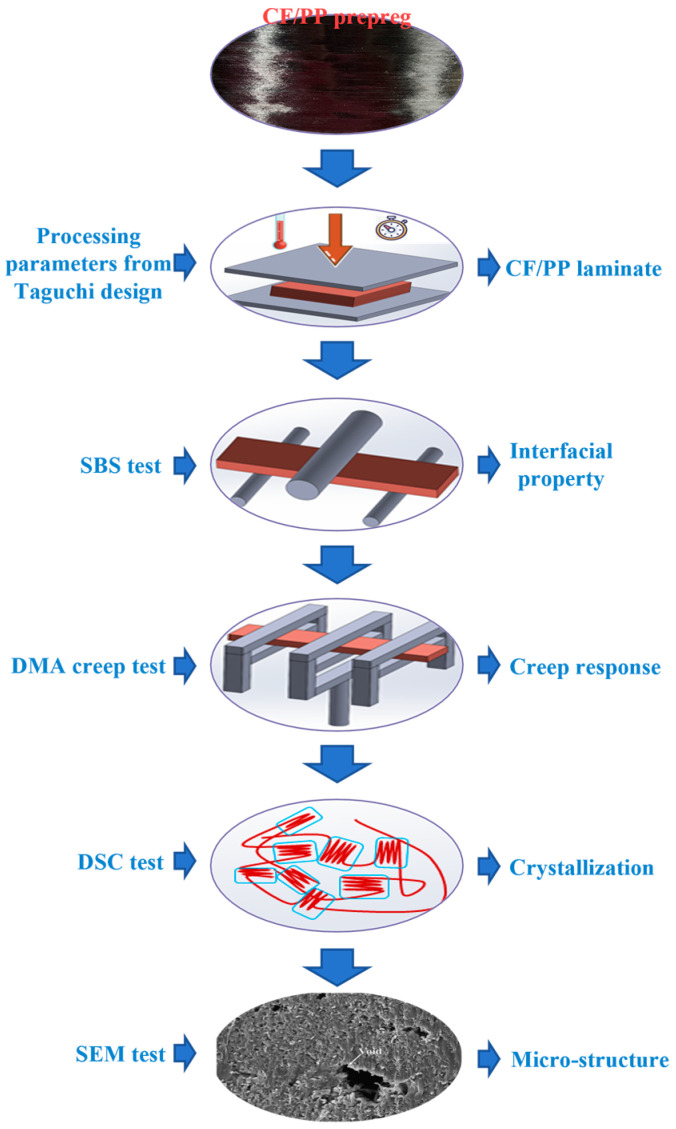
The flow chart of the main contents.

Given the research conditions mentioned above, the influence of processing parameters on the mechanical, thermo-mechanical and creep-recovery properties of continuously unidirectional CF/PP composites (produced from prepregs and the hot-press method) remains unclear. The present study aims to study the influence of molding temperature, pressure and holding time on the short beam strength, storage modulus, loss modulus, tan δ, creep strain, strain recovery rate and crystallinity degree through the Taguchi experiment design. The impregnation condition was observed by the scanning electron microscope test and correlated to the short beam strength of CF/PP composites. Figure 1 is the flow chart showing the main contents of the manuscript.

## 2. Experimental

### 2.1. CF/PP Prepreg

**Figure 2:**
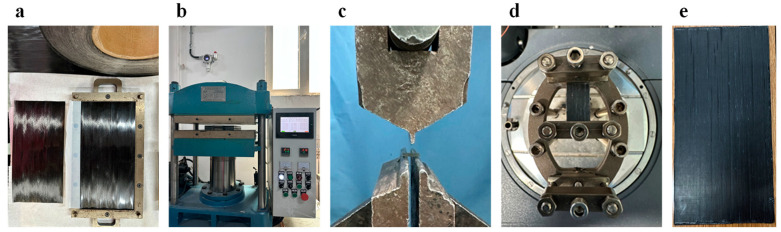
Experiment preparation including the (**a**) CF/PP prepreg, (**b**) hot-press processing, (**c**) short beam strength test, (**d**) dynamic mechanical analysis test for thermo-mechanical and creep-recovery behavior characterization of CF/PP composites, and (**e**) CF/PP laminates.

As the input materials of CF/PP composites, the unidirectional CF/PP prepreg (see Figure 2a) was purchased from Chengzi Tainuo (Shandong, China) New Material Technology Co., Ltd. (Tai’an, China), and its general properties are presented in Table 1. The tensile strength of CF/PP prepreg was also tested by the authors according to the standard ASTM D3039 [30]. The tensile test specimen has a length, width, and thickness of 350, 5 and 0.25 mm. The anchorage of the CF/PP prepreg was achieved by using the sandpaper, and the anchorage length is 50 mm at each end. The tensile test speed was set as 2 mm/min. The tensile strength of nine CF/PP prepreg specimens was 1398.8, 1360.6, 1167.0, 1134.1, 1448.7, 1314.5, 1231.9, 1383.1 and 1144.8 MPa, respectively, leading to a mean and standard deviation value of 1287.1 and 113.1 MPa. Table 2 summarizes the standard used in Section 2.

### 2.2. Fiber Volume Content Test

The fiber weight content (*w*_f_) of CF/PP prepregs was tested based on the ignition loss method according to the standard GB/T 44307-2024/ISO 22821: 2021. The test was conducted by using the thermal gravimetric analysis (TGA) machine (DiscoveryTGA5500, Waters, Milford, MA, USA). The test procedure is that under nitrogen, the specimen’s temperature was heated from room temperature to 650 °C with a ramp rate of 10 °C/min and kept for 10 min. Then the temperature is cooled down to 400 °C, and then the nitrogen is replaced with air. Under air and at 400 °C, the specimen was held for 15 min. Finally, the specimen was heated from 400 to 800 °C with a ramp rate of 10 °C/min and kept for 20 min at 800 °C. The fiber weight content, *w*_f_ and fiber volume content, *v*_f_, are calculated according to Equations (1) and (2).

(1) $$w_{f}=\frac{m_{1}-m_{2}}{m_{0}}\times 100$$

(2) $$v_{f}=\frac{w_{f}/\rho_{f}}{w_{f}/\rho_{f}+w_{m}/\rho_{m}}$$

where *m*_1_, *m*_2_, *m*_0_, *ρ*_f_, *w*_m_ and *ρ*_m_ are the mass of specimens after pyrolysis (g), mass of specimens after pyrolysis and combustion of carbon fibers (g), mass of original specimens (g), carbon fiber density (g/cm^3^), matrix weight content (%) and matrix density (g/cm^3^).

### 2.3. Manufacturing of CF/PP Composites

The unidirectional CF/PP composites were produced by the hot-press method (see Figure 2b) and the type plate vulcanizer (XLB-DQ, Qingdao, China) through the stacking of unidirectional CF/PP prepregs in the same direction. At first, nine CF/PP prepregs were put into a steel mold (300 × 150 mm), and then pressure and temperature were applied. The final thickness of CF/PP composites is controlled by the depth of the steel mold, and it is about 2 mm. Figure 2e presents the surface of CF/PP laminates (210-3-10), and the surficial stripes were caused by the shrinkage of polytetrafluoroethylene membranes on the top and bottom of CF/PP prepregs.

The Taguchi experiment was conducted to investigate the sensitivity of CF/PP composites’ short beam strength and tan δ value during the dynamic mechanical analysis test to different processing parameters. The Taguchi experiment has three controlled factors, namely the molding temperature, pressure and holding time, and each factor has three levels (determined by referring to references [23,24,35,36,37,38]), which are listed in Table 3, similar to that in [39]. For convenience, the specimen’s name was made up of the processing parameters. As an example, specimen 190-1-5 means the CF/PP specimen was manufactured under 190 °C, 1 MPa and held for 5 min. The signal-to-noise (S/N) ratio was used to measure the sensitivity of every parameter, and a higher S/N ratio means a smaller product variance compared with the target value. The mean square deviation (MSD) was also used to perform S/N ratio analysis by using the following equations [40]:

(3) $$S/N=-10L{\mathrm{og}}_{10}\left(MSD\right)$$

(4) $$MSD=\frac{1}{n}\sum_{i=1}^{N}\frac{1}{y_{i}^{2}}$$

where *n* and *y*_i_ are the number of tests and the result of the *i*th test.

### 2.4. Short Beam Strength (SBS) Test

The short beam strength (SBS) test applies a three-point bending load to a short-beam specimen, and the failure mode is dominated by interlaminar shear cracking rather than flexural fracture, which was used to reflect the fiber-matrix quality of manufactured CF/PP composites (Figure 2c). The SBS test was conducted according to the standard ASTM D2344/D2344M-22, and the specimen’s length and width are six times and two times its thickness. The experiment was conducted by a universal testing machine (DHY-10080, Shanghai, China), and the loading speed was set as 1 mm/min with five repeated specimens. The SBS could be calculated by Equation (5).

(5) $$F^{sbs}=0.75\times \frac{P_{m}}{b\times h}$$

where *F*^sbs^, *P*_m_, *b* and *h* are the SBS (MPa), the maximum load during the test (N), specimen width and specimen thickness (mm).

After getting the SBS of repeated CF/PP specimens, the one-way analysis of variance (ANOVA) method was adopted to assess whether there was a significant difference between the mean and standard deviation of the SBS of CF/PP specimens with the three processing parameters.

### 2.5. Dynamic Mechanical Analysis (DMA) Test

The thermo-mechanical properties of CF/PP composites were measured using the Q800 instrument (TA Instruments, New Castle, DE, USA) (see Figure 2d). CF/PP composite specimens were loaded in a single cantilever mode with an amplitude and a single frequency of 20 μm and 1 HZ. Specimens were heated from −40 to 140 °C with a ramp rate of 5 °C/min. One specimen was tested for every CF/PP laminate manufactured under different conditions.

Thermo-mechanical properties of CF/PP composites from DMA test results have three indices, including storage modulus, loss modulus and tan δ. The storage modulus and loss modulus, respectively, measure the ability of CF/PP composites to store and dissipate energy. The onset temperature of the storage modulus curve was considered as the glass transition temperature (*T*_g_) of CF/PP composites according to the specification in standard ASTM D7028-07 (Reapproved 2024). The tan δ value shows materials’ damping potential and could be calculated by Equation (6).

(6) $$\tan\delta =\frac{E^{\prime \prime }}{E^{\prime }}$$

where *E*″ and *E*′ are the loss modulus (MPa) and the storage modulus (MPa).

In addition, the creep-recovery behaviors of CF/PP composites were also tested by using the Q800 instrument. The applied stress of the creep-recovery test was set as 5 MPa, considering the maximum force of this kind of DMA equipment is only 18 N and the time for the creep and recovery process was set as 60 min. Two temperatures were applied to the creep-recovery test, including 30 and 80 °C.

### 2.6. Differential Scanning Calorimetry (DSC) Test

The preparation parameters could influence the microstructure of composites and therefore determine their properties [41]. The DSC test was conducted by the DiscoveryDSC2500 (Waters, USA) equipment and used to investigate the influence of processing parameters on the crystallinity behavior and degrees of the CF/PP composites, trying to explain the change in properties. Approximately 10 mg CF/PP powders were tested for each CF/PP composite from different manufacturing conditions. Then, under a nitrogen gas flow of 50 mL/min, specimens were rapidly heated from room temperature to 220 °C and held isothermally for 5 min to eliminate thermal history. Next, cooled specimens to 100 °C at a rate of 10 °C/min and held isothermally for 1 min. Subsequently, heated specimens again to 200 °C at a rate of 10 °C/min. The specimen from different manufacturing conditions was tested only once. The crystallinity degree of CF/PP composites was calculated according to Equation (7).

(7) $$X_{C}=\frac{H_{m}}{\left(1-m_{f}\right)\times H_{m}^{0}}\times 100\%$$

where *X_C_*, *H_m_*, *m_f_*, and *H_m_*^0^ are the crystallinity degree, melting enthalpy of the specimen (J/g), fiber weight content of the CF/PP specimen and the melting enthalpy of 100% crystalline specimen (165 J/g, taken from [42]).

### 2.7. Scanning Electron Microscope and Void Content Test

The scanning electron microscope (SEM) is an electron-optical device to characterize the surface microstructure and fracture morphology of materials at high magnification, and the test was conducted to investigate the influence of processing parameters on the manufacturing qualities of CF/PP composites. Cut small specimens out of CF/PP composites and polished the cross-section of the specimens. Then the ultrasonic treatment was applied to the specimens to remove small particles. The SEM test was conducted using the VEGA3 SEM (TESCAN, Brno, Czech Republic) equipment, and more operational details can be found in ref. [1].

The void content of CF/PP laminates was obtained according to the standard ASTM D2734-23. The CF/PP specimen has a shape of 35 × 35 × 2 mm^3^, larger than the required volume 2 cm^3^, specified in the standard and five repeats were prepared for each specimen produced from different manufacturing conditions. The void content could be calculated by Equation (8).

(8) $$\begin{array}{c} V=100\left(T_{d}-M_{d}\right)/T_{d}\\ T_{d}=100/\left(R/D+r/d\right) \end{array}$$

where *V*, *T*_d_, *M*_d_, *R*, *D*, *r* and *d* mean void content (volume, %), theoretical composite density (g/cm^3^), measured composite density (g/cm^3^), resin in composite (weight, %), density of resin (g/cm^3^), fiber in composite (weight, %) and fiber density (g/cm^3^).

## 3. Results and Discussion

This section mainly presents the results and discussion related to the above experiment listed in Section 2.

### 3.1. Fiber Volume Content of CF/PP Composites

**Figure 3:**
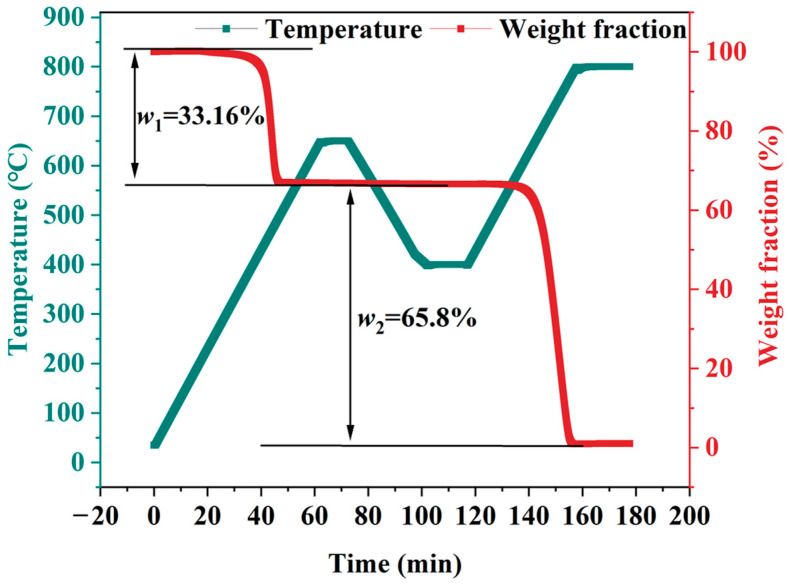
The TGA curve of CF/PP prepregs.

The TGA curve of CF/PP prepregs is listed in Figure 3. As seen, the figure demonstrates a distinct two-step thermal degradation process under aerobic conditions. The first mass loss of CF/PP prepregs occurred between 400 and 550 °C, corresponding to the thermal decomposition and volatilization of the PP matrix [43]. The subsequent mass loss in the range of 550 to 800 °C was attributed to the oxidative combustion of the carbon fiber, with nearly no residual mass at 800 °C. In summary, the mass lost due to PP pyrolysis *w*_1_ and mass lost due to carbon fiber combustion *w*_2_ are 33.16% and 65.8%. According to Equations (1) and (2), the fiber weight content and fiber volume content were calculated as 65.8% and 49.0% (density of carbon fiber and polypropylene matrix was set as 1.8 and 0.9 g/cm^3^ [44]).

The fiber content in this study was also compared with that in the literature. Kim and Lee [45] manufactured carbon fabric reinforced thermoplastic polypropylene composites with a fiber volume fraction from 48.52% to 55.01%, depending on the thickness of the interleaved film. Ueda et al. [46] achieved the 3D printing of a continuous carbon fiber reinforced blending polymer of PP and polyamide 6 with a fixed fiber volume fraction of 40%. These data indicate that the prepreg used in the present study has a reasonable fiber volume content.

### 3.2. SBS of CF/PP Composites

**Figure 4:**
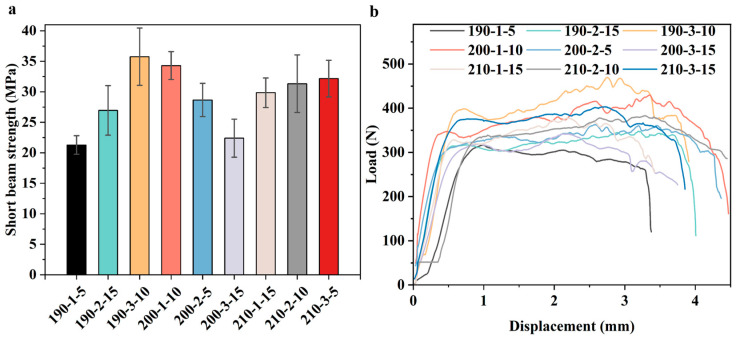
The (**a**) SBS and (**b**) load–displacement curves of CF/PP composites.

The SBS and load–displacement responses of unidirectional CF/PP composites systematically elucidate the effects of processing parameters on their interlaminar shear performance, as illustrated in the bar chart and load–displacement curves in Figure 4. The detailed SBS values of CF/PP composites produced under different processing parameters are listed in the second column of Table 4. As seen, the minimum and maximum SBS for CF/PP composites are 21.3 and 35.8 MPa (a variation rate of 68.1%), which clearly means that the processing parameters play a key role in the interfacial quality of CF/PP composites. Moreover, the SBS value of CF/PP composites obtained in this study is reasonable compared with that in other studies. As an example, the maximum SBS for CF/PP composites made from filament winding could reach 16.37 MPa [47]. The maximum interlaminar shear strength of polyetherimide nanoparticles modified CF/PP composites could reach 27.66 MPa [48]. The maximum shear strength of woven CF/PP composites was around 8.75 MPa, reported by Sun et al. [24].

A cross-parameter correlation analysis revealed that the molding temperature interacted strongly with pressure and holding time. For instance, at low pressure (1 MPa), the influence of holding time was relatively limited, and SBS values remained at a moderate level regardless of temperature variation. However, under high pressure (3 MPa), the effect of holding time became pronounced, especially at elevated temperatures. For example, the combination of high pressure, high molding temperature, and long holding time led to severely decreased SBS of CF/PP composites, which implies a synergistically negative effect. Over-pressure tended to cause matrix depletion inside fiber tows, while prolonged heating accelerated thermal oxidation. Together, these factors aggravate interfacial defects and stress concentrations. In comparison, the combination of moderate temperature (190 °C), appropriate pressure, and medium holding time (10 min) yielded the highest SBS. This optimized condition ensured sufficient melt flow of polypropylene matrix and complete impregnation of carbon fibers. The error bars further confirmed that suitable parameter combinations improved the experimental stability and repeatability of the SBS test.

Figure 4b presents the load–displacement curves of all CF/PP composites. As seen, the load–displacement curves for CF/PP composites produced under different processing parameters have the same shapes. The initial section of the load–displacement curve was straight, and then there was a non-linear plateau, followed by a rapid drop, which is a typical load–displacement curve for 0° carbon fiber reinforced polymer composites [49]. In addition, specimens with higher SBS exhibited a longer non-linear plateau and larger failure displacement, indicating ductile interface failure with effective energy absorption. In contrast, low-strength specimens showed abrupt failure after the elastic stage, typical of brittle interfacial debonding.

**Figure 5:**
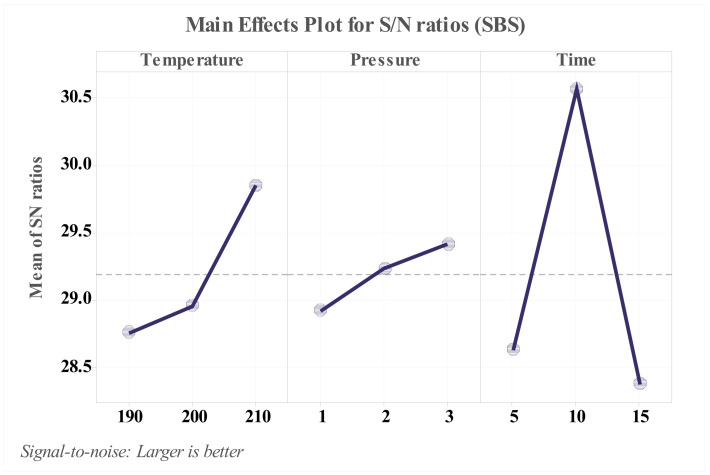
Main effect plot for S/N ratios regarding SBS.

The S/N ratio main-effects plot quantifies the sensitivity of the CF/PP composite’s SBS to each processing parameter and enables cross-parameter correlation analysis, which is referred to in Table 5 and Figure 5. According to the biggest delta of the S/N ratio values, the holding time has the biggest influence on the SBS of CF/PP composites, followed by the temperature and finally the pressure.

The main-effects plot also reveals the strong cross-parameter correlations. The optimal combination derived from S/N analysis included a moderate temperature, relatively low pressure, and medium holding time. This optimal combination could achieve balanced matrix fluidity, sufficient impregnation, and structural stability. However, severe mismatch among parameters might induce defects like voids, unmelt matrix, and thermal decomposition of matrix and sizing agents of fibers. Therefore, the SBS of CF/PP composites is determined not only by one individual parameter but more importantly by their synergistic match, which could guide the performance improvement of manufactured CF/PP composites.

The ANOVA results for the SBS of CF/PP laminates were presented in Table 6. As seen, the result indicated that the holding time is the most significant factor (*p* value is much less than 0.05) and plays a key role in the SBS of CF/PP laminates, which is consistent with the result obtained by the main effect plot for the S/N ratios method. The main effects of temperature and pressure were not obvious in this experiment, indicating that their individual effects on SBS were relatively minor.

### 3.3. Thermo-Mechanical Property Results of CF/PP Composites

**Figure 6:**
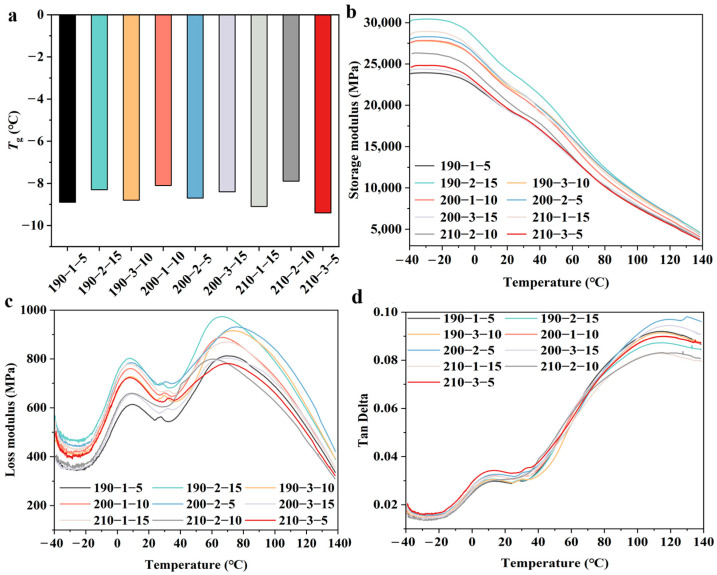
DMA results of CF/PP composites produced under different situations, including (**a**) *T*_g_, (**b**) storage modulus, (**c**) loss modulus and (**d**) tan *δ*.

The thermo-mechanical results obtained by the DMA test of CF/PP composites are shown in Figure 6, which are similar to the test results regarding CF/PP composites obtained from other studies [50,51]. The *T*_g_ of CF/PP composites produced under different situations was listed in the third column of Table 4 and Figure 6a. As seen, the *T*_g_ of all nine types of CF/PP composites is negative, which is in accordance with the *T*_g_ of the PP matrix published by other studies, like −10 to 10 °C by Thomason and Yang [52]. In addition, it is seen that the production situations have relatively limited influence on the *T*_g_ of CF/PP composites, and the variation rate of *T*_g_ for nine CF/PP composites is 16.0%.

As seen in Figure 6b, processing parameters have an obvious influence on the storage modulus of CF/PP composites. Generally, a higher storage modulus means a better adhesion between carbon fiber and polypropylene matrix, which was reported by Luo et al. [51]. Although the test results in this study were not entirely consistent with this conclusion, partial results followed this point. For instance, four lines with higher storage modulus values were CF/PP specimens 190-2-15, 210-1-15, 200-2-5, and 200-1-10, and they had an SBS of 27.0, 29.9, 28.7 and 34.3 MPa, respectively, while the CF/PP specimen with the lowest storage modulus had the lowest SBS of 21.3 MPa. The variation rate of the peak storage modulus is 27.2%. The temperature corresponding to the peak loss modulus ranged from −35.7 to −29.0 °C with a variation rate of 18.7%.

Processing parameters also sharply affect the loss modulus of CF/PP composites, as seen in Figure 6c. The first peak between 0 and 20 °C was also observed by Luo et al. [51], but the second peak did not appear in this study. The first peak corresponds to the *T*_g_ of PP based on the temperature data, and the second peak indicates the ability of energy dissipation of CF/PP composites. The peak values of loss modulus curves ranged from 781.1 to 973.5 MPa for CF/PP composites produced under different manufacturing conditions. The variation rate of the peak loss modulus is 24.6%. The temperature corresponding to the peak loss modulus ranged from 60.6 to 75.1 °C with a variation rate of 23.9%. The biggest loss modulus of CF/PP composites was obtained under the manufacturing conditions of 190 °C, 2 MPa and 15 min.

Figure 6d presents that the processing parameters also play a key role in the variation in tan *δ* value of CF/PP composites. A similar shape was also reported by Luo et al. [51] and Yeole et al. [53] and the first peak appeared between 0 and 20 °C. The second peak in Figure 6d was not reported by Luo et al. [51] and Yeole et al. [53] because they ended the DMA experiment at 100 °C. The peak tan *δ* value had a range from 0.083 to 0.098, and the temperature at the peak of tan *δ* ranged from 110 to 130 °C. The biggest tan *δ* value of CF/PP composites was obtained at the manufacturing conditions of 200 °C, 2 MPa and 5 min.

**Figure 7:**
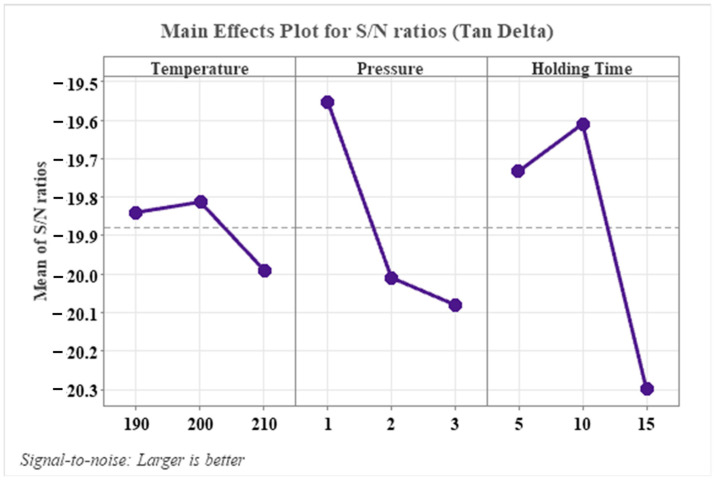
Main effect plot for S/N ratios regarding the tan *δ* of CF/PP composites.

The main effects plot for the signal-to-noise (S/N) ratios of tan *δ* for CF/PP composites illustrates the critical influence of processing parameters on their damping performance, referring to Table 7 and Figure 7. As seen in the table, the D-value between the maximum and minimum S/N ratio values was 0.18, 0.53 and 0.69 for parameters molding temperature, pressure, and holding time, which means the holding time was the most influential parameter, followed by pressure and temperature. Moreover, moderate molding temperature (200 °C), moderate pressure (1 MPa), and optimal holding time (10 min) yielded the highest tan *δ* S/N ratios, reflecting balanced interfacial adhesion (SBS of 34.3 MPa at this condition) and structural integrity that maximizes the composite’s damping performance.

### 3.4. Creep-Recovery Results of CF/PP Composites

**Figure 8:**
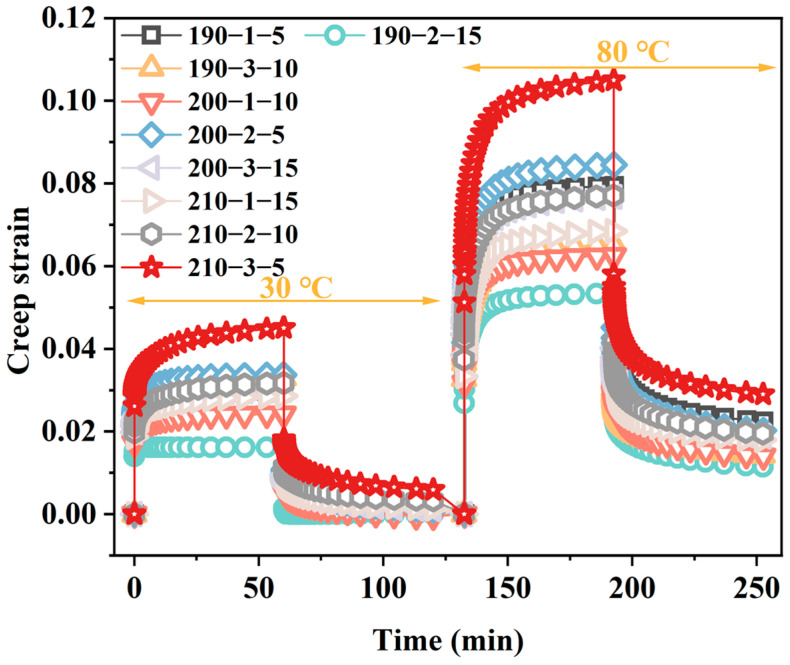
The creep-recovery curves for CF/PP composites manufactured at different conditions.

The creep-recovery curve for different CF/PP composites was plotted in Figure 8. As illustrated in the creep strain curves, CF/PP composites under different conditions exhibited similarly typical viscoelastic behavior. In detail, at first, for the creep curve, the flexural strain increased instantly after loading and accumulated progressively under sustained loading at both temperatures, followed by partial strain recovery upon load removal. Notably, creep deformation was significantly amplified at 80 °C compared to 30 °C, but the creep recovery rate was obviously reduced, which means more molecular chain mobility occurred at elevated temperatures. The specimen 210-3-5 demonstrated the highest maximum creep strain (0.1% at 80 °C), while the CF/PP specimen 190-2-15 showed the lowest (0.053% at 80 °C). The detailed maximum creep strain and the creep-strain recovery rate at two temperatures are referred to in Table 8.

The histograms of strain recovery rate for CF/PP composites further quantify the viscoelastic recovery capacity, referring to Figure 8. At 30 °C, all composites maintained high recovery rates (87–102%), with 190-2-15 and 200-1-10 achieving near-complete recovery (>100%), reflecting excellent elastic resilience. In contrast, recovery rates of CF/PP composites at 80 °C dropped sharply to 71–79%, attributed to enhanced matrix softening and irreversible viscous flow at elevated temperatures.

**Figure 9:**
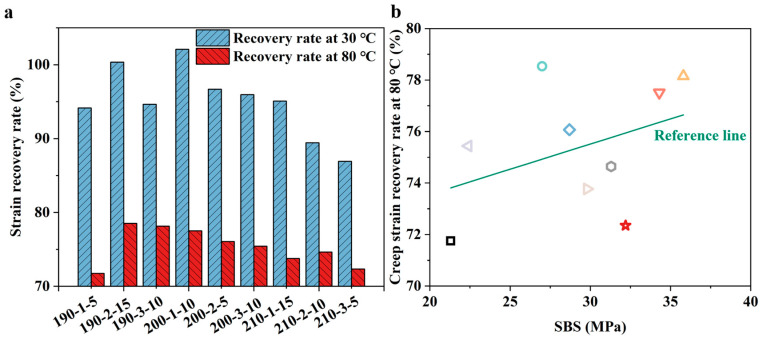
The creep recovery rate (**a**) at 30 and 80 °C for CF/PP composites and (**b**) compared with the SBS of CF/PP composites.

In general, a better fiber-matrix interface leads to a better creep resistance behavior [54,55] for FRTP composites because the good interfacial bond takes advantage of the low deformation ability of reinforcing fibers by load transferring. However, when comparing the maximum creep strain or the creep strain recovery rate with the SBS of the nine CF/PP composites (Figure 9b), the mapping relationship between them is complex. Notably, there was a rough negative correlation between the SBS and the creep resistance, especially when considering the lowest and the largest SBS. For instance, specimen 190-1-5 had the lowest SBS of 21.3 MPa and the lowest creep strain recovery rate, while specimens 190-3-10 and 200-1-10 exhibited the highest SBS and the largest creep strain recovery rate, which could be explained by the fact that better adhesion could restrict the mobility of polymer chains.

### 3.5. Crystallinity Behavior of CF/PP Composites

**Figure 10:**
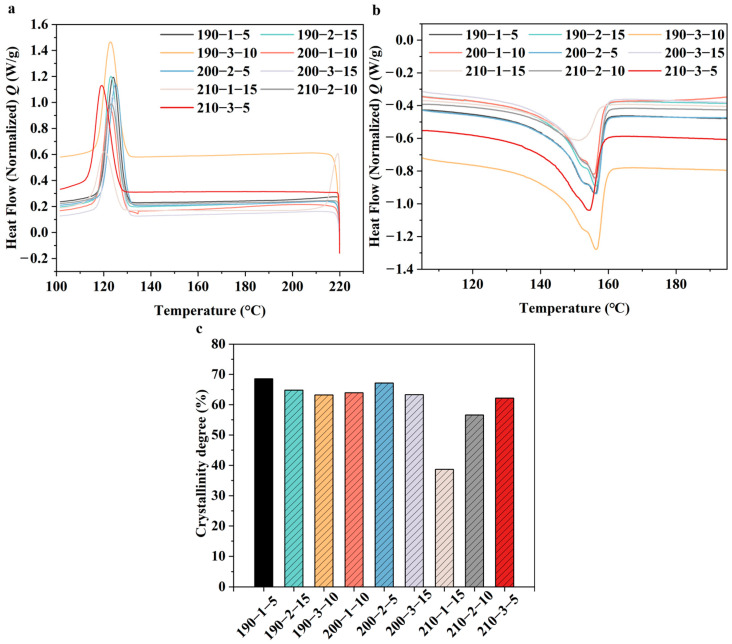
The DSC curves of (**a**) cooling, (**b**) second heating stage and (**c**) crystallinity degrees for CF/PP composites under different processing conditions.

DSC cooling curves of different CF/PP laminates after erasing the previous thermal history are shown in Figure 10a. All CF/PP laminates exhibited a single crystallization exotherm with peak temperatures ranging from 119.38 to 125.31 °C, corresponding to the non-isothermal melt crystallization of the PP matrix during cooling. No additional crystallization peak was observed, indicating that the hot-pressing parameters did not change the fundamental crystallization pathway of the PP matrix, but mainly affected its crystallization kinetics. The variation in crystallization peak temperature and peak shape suggests that the molding temperature, pressure, and holding time jointly influenced the chain rearrangement and heterogeneous nucleation behavior of the PP matrix when carbon fibers existed. Among the investigated specimens, 200-2-5 showed a relatively higher crystallization peak temperature, implying that crystallization occurred at a lower degree of supercooling. In contrast, specimens like 210-3-5 and 210-1-15 exhibited crystallization peaks at lower temperatures and/or broader peak profiles, suggesting delayed crystallization and slower crystallization kinetics.

The second heating process curves of the DSC test were illustrated in Figure 10b because the first heating process was used to eliminate the thermal stress. As illustrated, the normalized DSC heating curves revealed distinct melting behaviors across CF/PP composites produced from different conditions, with variations in melting peak position, peak intensity, and endothermic enthalpy directly reflecting differences in crystallinity and crystal perfection. The corresponding crystallinity degree (quantified in Figure 10c and the fourth column of Table 4) of CF/PP composites demonstrated a wide range from 38.65% to 68.5%, highlighting the profound impact of processing parameters on the crystalline structure of the polypropylene matrix. Moreover, the crystallinity degrees of CF/PP composites used in the present study were also compared with the values reported in other studies. For instance, Liu et al. [50] reported the crystallinity degrees of CF/PP composites during the second heating process were 51.2% to 59.5%, considering different modification methods. Sun et al. [24] reported that the crystallinity degrees of CF/PP composites were 56.37%, 63.43% and 66.25%, considering different stamping temperatures, including 190, 220, and 250 °C. Tian et al. [27] presented the crystallinity degrees of pure PP and PP reinforced with different contents of carbon fiber and carbon black, and their crystallinity degrees were above 50%. In more detail, the crystallinity degrees of CF/PP composites increased with the contents of carbon fiber. Huang et al. [56] found the crystallinity degrees of CF/PP composites with a fiber weight content below 20% were around 40%, and fiber weight contents did not influence their crystallinity degrees. In conclusion, the crystallization degrees obtained in the present study were slightly higher than the reported values in the literature, which might be attributed to the higher fiber weight content in the CF/PP composites used by the authors.

**Figure 11:**
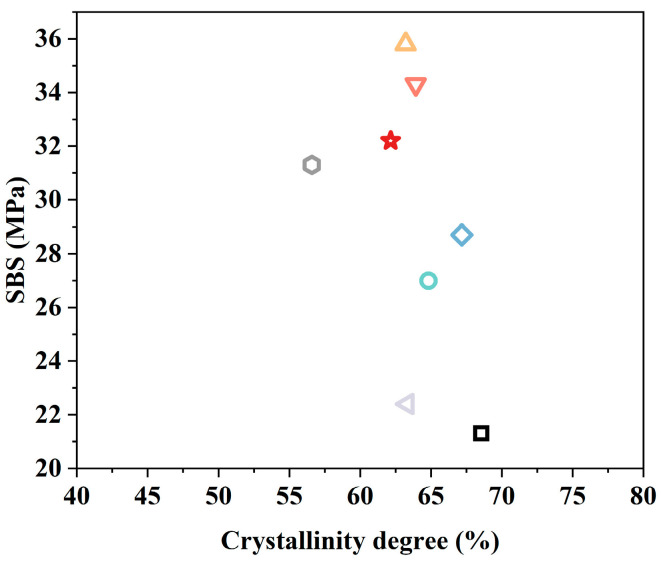
The relationship between crystallinity degrees and SBS of CF/PP composites.

Notably, a clear negative correlation is observed between the crystallinity degree and SBS of CF/PP composites (Figure 11), where specimens with lower crystallinity (e.g., 210-1-15, 38.65%) exhibited higher SBS values (29.9 MPa), while those with ultra-high crystallinity (e.g., 190-1-5, 68.5%) showed significantly reduced interfacial strength (21.3 MPa). This phenomenon originated from the competing effects of processing parameters on matrix crystallization and interfacial bonding within CF/PP composites. In detail, elevated molding temperature, prolonged holding time, and optimized pressure could promote the interfacial wetting of carbon fibers, but simultaneously might disrupt the regular packing of PP’s molecular chains, suppress heterogeneous nucleation at the fiber-matrix interface, and reduce the overall crystallinity degree of CF/PP composites.

Furthermore, the DSC curves of different CF/PP composites revealed that specimens with a higher crystallinity degree exhibited sharper and higher melting peaks, indicating the formation of more perfect crystalline structures, while those with lower crystallinity showed broader, lower-intensity peaks, characteristic of a more amorphous matrix with smaller, defective crystals. This microstructural difference in different CF/PP composites could correlate to their previous creep-recovery behaviors. For instance, low-crystallinity CF/PP specimens with a more amorphous matrix and strong interfacial bonding exhibit enhanced viscoelastic recovery rate while high-crystallinity specimens possess a rigid, glassy matrix at room temperature, leading to reduced elastic recovery, as the crystalline regions restrict molecular chain mobility.

### 3.6. SEM and Void Content Results of CF/PP Composites

**Figure 12:**
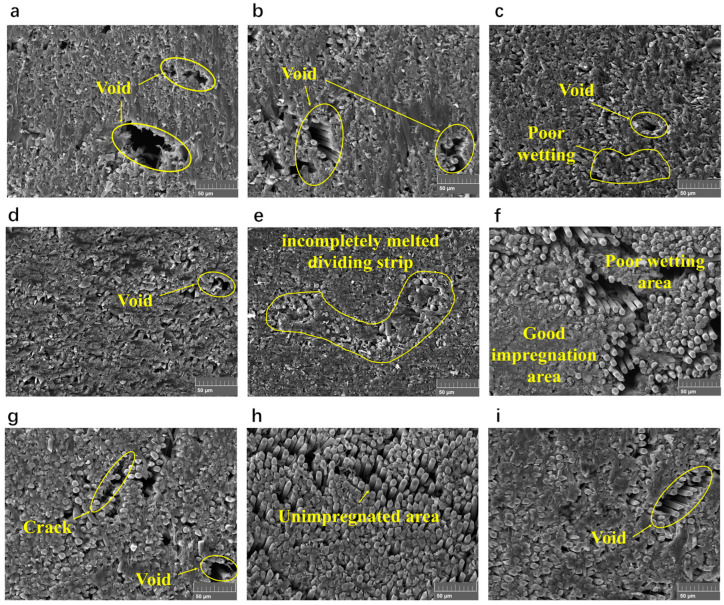
SEM of the cross-section for CF/PP composites produced under (**a**) 190-1-5, (**b**) 190-2-15, (**c**) 190-3-10, (**d**) 200-1-10, (**e**) 200-2-5, (**f**) 200-3-15, (**g**) 210-1-15, (**h**) 210-2-10 and (**i**) 210-3-5.

Figure 12 shows the cross-section of CF/PP composites from nine different manufacturing conditions. As illustrated, specimens with different SBS values exhibited relatively distinct microstructural characteristics. Generally, all nine CF/PP specimens have void defects, which could serve as stress concentration points to reduce their interfacial properties. Moreover, there were other defects, including incompletely melted dividing strip (Figure 12e), poor wetting area (Figure 12c,f) and unimpregnated area (Figure 12h), and these defects could result in failures along these areas, influencing the internal stress transferring within the CF/PP laminates. These voids and poor wetting-related defects could both be attributed to the high viscosity of the PP matrix because the high viscosity could trap air during the hot-press and make the flow of resin within the fibers extremely difficult. The existence of all these defects within CF/PP laminates makes their SBS much lower than the unidirectional carbon fiber reinforced epoxy laminates (minimum value could range from 43.9 to 80.7 MPa) [57,58,59].

**Figure 13:**
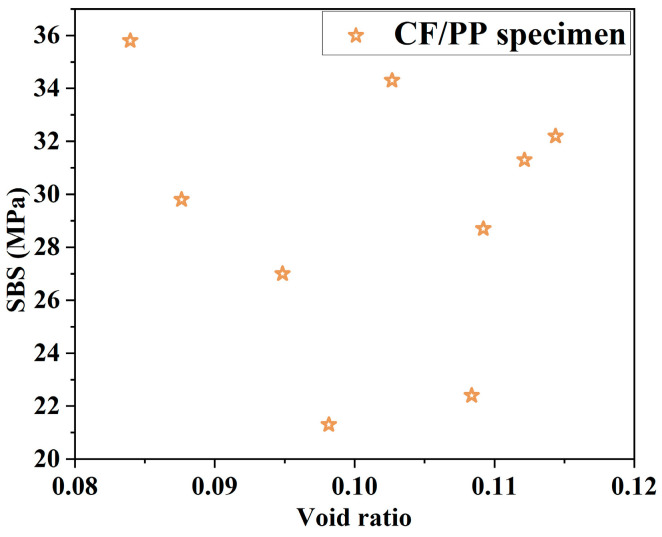
SBS and void content of different CF/PP specimens.

The void content versus SBS value curve of CF/PP laminates produced from different manufacturing conditions is shown in Figure 13. The void content of the CF/PP laminates was relatively high, which might contribute to the high volume content of carbon fibers [60]. As seen, although the largest CF/PP specimen had the lowest void content, no clear linear negative correlation is observed, indicating that void content (8.4%) alone cannot fully explain the variation in SBS. Instead, significant data scatter suggests that other factors, such as fiber-matrix interfacial bonding quality, crystallization degree and matrix property strongly influence the mechanical performance, even at similar void contents.

## 4. Conclusions

The present study investigated the influence of processing parameters on the short-beam strength (SBS), thermo-mechanical properties, creep-recovery behavior and crystallinity degrees of CF/PP composites based on the Taguchi experiment design. Moreover, the cross-section was observed by the SEM test. The following conclusions could be drawn.

Processing parameters, including molding temperature, pressure and holding time, have an obvious influence on the internal bonding of CF/PP composites. An appropriate combination of processing parameters could increase their SBS by 59.8% and reduce the void content by 2.4% (Specimens 190-3-10 and 200-3-15). The SEM analysis presented that the relatively low SBS of CF/PP composites comes from void and poor fiber impregnation problems, such as incomplete melted matrix.

In addition, processing parameters have relatively limited effects on the glass transition temperature of CF/PP composites (−7.9 to −9.4 °C) but have obvious impacts on their peak storage and loss modulus value (variation rate of 27.2% and 24.6%) during the DMA test. High service temperatures (up to 80 °C) reduced the creep recovery rate to 71–79%, suggesting careful consideration of operating conditions for long-term applications. Moreover, there was a rough positive correlation between the SBS and creep strain recovery rate at 80 °C of CF/PP composites.

Processing parameters could determine the crystalline behavior of CF/PP composites. Through a suitable choice of processing parameters, the variation in crystallinity degree could reach 77.36%. CF/PP composites with higher crystallinity degrees exhibited a sharper and higher melting peak, an index of a more perfect crystalline structure. There is a strong negative relationship between the SBS and crystallinity degrees of CF/PP composites.

The study provides quantitative guidance for manufacturing industrial CF/PP composite for structural applications. Future work could focus on scale-up effects and application-specific optimization of mechanical and thermo-mechanical properties of CF/PP composites.

## Figures and Tables

**Table 1:** General properties of CF/PP prepregs from the producer.

Width (mm)	Thickness (mm)	Tensile Strength (MPa)	Tensile Modulus (GPa)	Elongation at Break (%)	Areal Density (g/m^2^)
150	0.25	1100	72	1.62	310

**Table 2:** The standard and test speed of different properties for CF/PP prepregs and composites.

Test Name	Standard	Test Speed
Tensile test of CF/PP prepreg	ASTM D3039/D3039M-17 Standard test method for tensile properties of polymer matrix composite materials	2 mm/min
Fiber weight content test of CF/PP prepreg	GB/T 44307-2024/ISO 22821: 2021 Carbon-fiber-reinforced composites—Determination of fiber weight content—Thermogravimetry (TG) [31]	/
Short beam strength test of CF/PP laminates	ASTM D2344/D2344M-22 Standard Test Method for Short-Beam Strength of Polymer Matrix Composite Materials and Their Laminates [32]	1 mm/min
Glass transition temperature of CF/PP laminates	ASTM D7028-07 (Reapproved 2024) Standard Test Method for Glass Transition Temperature (DMA Tg) of Polymer Matrix Composites by Dynamic Mechanical Analysis (DMA) [33]	/
Void content of CF/PP laminates	ASTM D2734-23 Standard Test Methods for Void Content of Reinforced Plastics [34]	/

**Table 3:** The experimental design for manufacturing CF/PP composites.

Experiment Number	Processing Parameters		
	Molding Temperature (°C)	Pressure (MPa)	Holding Time (min)
1	190	1	5
2	190	2	15
3	190	3	10
4	200	1	10
5	200	2	5
6	200	3	15
7	210	1	15
8	210	2	10
9	210	3	5

**Table 4:** SBS, glass transition temperature and crystallinity degree of CF/PP composites manufactured under different conditions.

Specimen Number	SBS/MPa	*T*_g_/°C	Crystallinity Degree/%
190-1-5	21.3	−8.9	68.55
190-2-15	27.0	−8.3	64.82
190-3-10	35.8	−8.8	63.21
200-1-10	34.3	−8.1	63.92
200-2-5	28.7	−8.7	67.18
200-3-15	22.4	−8.4	63.33
210-1-15	29.9	−9.1	38.65
210-2-10	31.3	−7.9	56.59
210-3-5	32.2	−9.4	62.15

**Table 5:** S/N ratio of SBS of CF/PP composites based on the larger-is-better principle.

Level	Temperature	Pressure	Holding Time
1	28.8	28.9	28.6
2	29.0	29.2	30.6
3	29.9	29.4	28.4
Delta	1.1	0.5	2.2
Rank	2.0	3.0	1.0

**Table 6:** The results of ANOVA for SBS.

Source of Variation	Degree of Freedom	Adj SS	Adj MS	F Ratio	*p* Value
Temperature	28.8	71.87	35.934	1.71	0.192
Pressure	29.0	13.16	6.578	0.31	0.732
Time	29.9	526.87	263.433	12.56	0.00005
Error	43	901.53	20.966	/	/
Total	49	1529.42	/	/	/

**Table 7:** S/N ratio of tan *δ* of CF/PP composites based on the larger-is-better principle.

Level	Temperature	Pressure	Holding Time
1	−19.84	−19.55	−19.73
2	−19.81	−20.01	−19.61
3	−19.99	−20.08	−20.30
Delta	0.18	0.53	0.69
Rank	3	2	1

**Table 8:** Maximum flexural strain and strain recovery rate for CF/PP composites under different conditions at 30 and 80 °C.

Specimen Number	30 °C		80 °C	
	Strain	Strain Recovery Rate (%)	Strain	Strain Recovery Rate (%)
190-1-5	0.030	94.15	0.079	71.75
190-2-15	0.016	100.36	0.053	78.53
190-3-10	0.033	94.65	0.065	78.15
200-1-10	0.024	102.10	0.063	77.51
200-2-5	0.034	96.69	0.085	76.07
200-3-15	0.030	95.95	0.077	75.44
210-1-15	0.029	95.08	0.068	73.77
210-2-10	0.032	89.43	0.077	74.64
210-3-5	0.045	86.93	0.100	72.35

## Data Availability

Data are contained within the article. Further inquiries can be directed to the corresponding author.
